# Circulating free heme induces cytokine storm and pulmonary hypertension through the MKK3/p38 axis

**DOI:** 10.1152/ajplung.00422.2022

**Published:** 2024-08-28

**Authors:** Mathews Valuparampil Varghese, Joel James, Dinesh Bharti, Franz Rischard, Olga Rafikova, Ruslan Rafikov

**Affiliations:** ^1^Division of Pulmonary, Critical Care, Sleep, and Occupational Medicine, Department of Medicine, Indiana University, Indianapolis, Indiana, United States; ^2^Department of Medicine, The University of Arizona College of Medicine – Tucson, Tucson, Arizona, United States

**Keywords:** cytokine storm, free heme, p38 kinase, pulmonary hypertension, vascular remodeling

## Abstract

Hemolysis is associated with pulmonary hypertension (PH), but the direct contribution of circulating free heme to the PH pathogenesis remains unclear. Here, we show that the elevated levels of circulating free heme are sufficient to induce PH and inflammatory response in mice and confirm the critical role of mitogen-activated protein kinase kinase-3 (MKK3)-mediated pathway in free heme signaling. Following the continuous infusion of heme for 2 wk, wild-type (WT) but not MKK3 knockout (KO) mice develop PH, as evidenced by a significantly elevated right ventricular (RV) systolic pressure, RV hypertrophy, and pulmonary vascular remodeling. The MKK3/p38 axis, markedly activated by heme infusion in WTs, results in upregulated proliferative/cytokine signaling targets Akt, ERK1/2, and STAT3, which were abrogated in MKK3 KO mice. Moreover, the MKK3 KOs were protected against heme-mediated endothelial barrier dysfunction by restoring the tight junction protein zonula occludens-1 expression and diminishing the inflammatory cell infiltration in the lungs. Plasma cytokine multiplex analysis revealed a severe cytokine storm already 24 h after initiation of heme infusion, with a significant increase of 19 cytokines, including IL-1b, IL-2, IL-6, IL-9, and TNF-a, in WT animals and complete attenuation of cytokine production in MKK3 KO mice. Together, these findings reveal a causative role of circulating free heme in PH through activating inflammatory and proliferative responses. The central role of MKK3 in orchestrating the heme-mediated pathogenic response supports MKK3 as an attractive therapeutic target for PH and other lung inflammatory diseases linked to hemolytic anemia.

**NEW & NOTEWORTHY** This study demonstrates that elevated levels of circulating free heme can induce pulmonary hypertension (PH) and inflammation in mice. Continuous heme infusion activated the MKK3/p38 pathway, leading to increased right ventricular pressure, right ventricular hypertrophy, and vascular remodeling. This activation upregulated signaling cascades such as Akt, ERK1/2, and STAT3, whereas MKK3 knockout mice were protected against these changes and had reduced inflammatory responses, highlighting MKK3’s potential as a therapeutic target for PH.

## Graphical abstract

**Figure:**
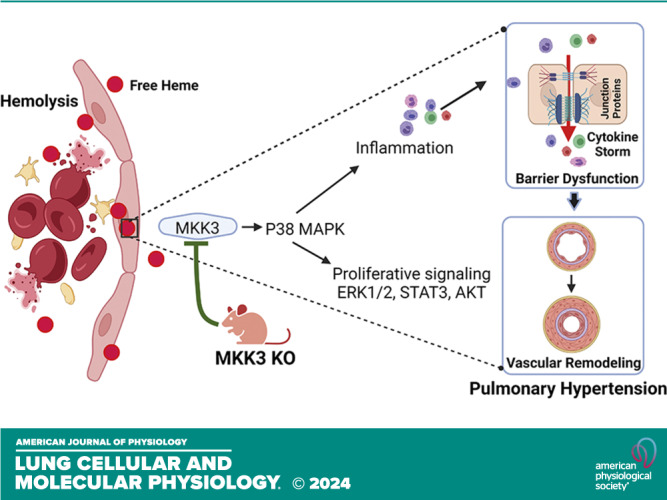

## INTRODUCTION

Hemolytic conditions are often associated with increased pressure in the pulmonary circulation. Thus, ∼10% of patients with chronic hemolytic anemia secondary to sickle cell disease (SCD) have pulmonary hypertension (PH; 1). This prevalence is considerably higher compared with the general population (2). Furthermore, PH was confirmed to be a significant risk factor for death in patients with SCD (3). Nevertheless, the PH secondary to hemolytic disorders (4) was recently moved in WHO classification from Group 1 PH to Group 5, which consists of conditions causing PH by unclear or multifactorial mechanisms. The inclusion of hemolytic PH patients in this group highlights the unmet need to understand the mechanisms underlying this type of PH, which, in turn, delays the development of adequate treatment approaches.

Notably, our recent study discovered the underlying hemolytic events in Group 1 idiopathic pulmonary arterial hypertension (PAH) patients and confirmed the correlation between hemolysis intensity and the severity of PAH (5). This finding suggests that classical hemolytic anemias and mild subclinical hemolytic events could contribute to the pathogenesis of PH. Nevertheless, what components of ruptured red blood cells (RBC) are responsible for pulmonary pressure increase and what mechanisms are involved in the pathobiology of PH remain unclear. The primary attention focuses on the abundant RBC protein—hemoglobin, which can scavenge nitric oxide and induce oxidative stress in the circulation (6). In hypoxic conditions, long-time elevated hemoglobin can affect pulmonary arterial pressure (7). However, although considered a pathological molecule, the free heme, a product of hemoglobin degradation, was not previously evaluated as a PH-driving agent.

In previous publications, we described that free heme could acutely disrupt endothelial barrier function and induce perivascular edema, local hypoxia, and subsequent pulmonary vascular remodeling (5, 6). One of the essential intracellular pathways for free heme-induced signaling was the mitogen-activated protein kinase kinase-3 (MKK3)/p38 axis. However, whether this heme-mediated pathway plays a causative role in the PH development rather than contributes to the complexity of the already developed disease remains unclear. In the present study, we continuously injected the free heme into the circulation of mice for 2 wk. This approach allowed us to delineate free heme’s contribution to the pathobiology of PH directly. To investigate the role of the MKK3/p38 axis in heme-mediated processes, we compared the effects of heme treatment in the wild-type (WT) and MKK3 knockout (KO) mice. The discovered pathogenic role of free heme, sufficient to induce PH phenotype in WTs, confirms that heme acts as a critical signaling molecule in PH. Furthermore, the protection revealed in MKK3 KO mice suggests that MKK3 is an important therapeutic target that could prevent the pathogenic effects of heme in severe and mild hemolytic conditions.

## METHODS

### Animal Model

This study used male 15-wk-old B6;129S1-Map2k3tm1Flv/J (MKK3 KO) and wild-type (WT) mice as previously published (8). Recently, we reported that, compared with female subjects, males are more susceptible to hemolysis and PH development (4). All the animals were housed at a temperature of 22°C, a 12-h light-to-dark cycle, and had free access to standard rodent food and water ad libitum. The University of Arizona Institutional Animal Care and Use Committee (IACUC) sanctions all experimental animal protocols. Animals were genotyped using the following primers: MKK3_F_FAM [6-FAM]- CCTGGAGCACCTGCATAGCAAG; MKK3_WT_R GGAGAGATGGCTCAGTGGTTAAAGC; MKK3_MUT_R CCAGCTCATTCCTCCACTCATG by University of Arizona Genetics Core, Tucson, AZ. Animals were grouped into four: Group I—WT vehicle, MKK3 KO vehicle, WT Heme, and MKK3 KO Heme (*n* = 8–10/group). The heme was continuously pumped into the blood circulation for 2 wk at the rate of 0.85 µg/h using ALZET osmotic minipumps (1004, Alza, Palo Alto, CA). For this purpose, the osmotic minipumps implanted under the skin of the neck were connected to the Mouse Jugular Catheter (0007700, DURECT Corporation, ALZET Osmotic Pumps, Cupertino, CA) catheter inserted into the jugular vein. At the end of the surgery, all group lungs were flushed with 5 mL of normal saline from the right heart, and the left lobe was immediately separated and transferred to buffered formalin. After 1 wk, they were moved to 70% ethanol for histological processing. Blood, lung, and heart tissues collected were stored at −80°C for further analysis. In additional experiments, mice were injected with a bolus dose of heme (250 µM) through the tail vein, and plasma samples were collected 24 h later.

### Hemodynamic Measurements

Mice were anesthetized with Ketamine (Zetamine, 501072, MWI, Boise, ID) (100 mg/kg) + Xylazine (AnaSed, 510004, MWI, Boise, ID) (10 mg/kg ip); Right ventricular systolic pulmonary pressure (RVSP) was evaluated using a pressure transducer catheter (SPR-1000, Millar Instruments, Houston, TX) connected to the Millar Transducer Control Unit TC-510 and PL3504 Power Lab 4/35 data acquisition system (ADInstruments, Colorado Springs, CO). The RVSP was measured by advancing the catheter through the right jugular vein into the right ventricular (RV) cavity. At the end of the pressure recording, the animals were attached to the mice ventilator system (MiniVent, Type-845; Harvard Apparatus, South Natick, MA). From the opened thorax, the lungs were flushed with a 0.9% NaCl solution. The lungs and heart were separated from the animals, and the left lung was used to fix in buffered formalin solution for histological studies. The right lung was stored at −80°C for other analyses. RV free wall was separated from the left ventricle with the septum (LV + S) and weighed. Fulton index (RV/LV + S ratio) was assessed as a measure of RV hypertrophy.

### Human Subjects

Deidentified lung samples consisted of Group 1-PAH patients [idiopathic pulmonary arterial hypertension (IPAH) group (*n* = 6)] and healthy controls group (*n* = 6). The samples were acquired through the Pulmonary Hypertension Breakthrough Initiative (PHBI). The PHBI study protocol was approved by the Institutional Review Boards of the participating sites in the network. All sites adhered to the requirements of the U.S. Federal Policy for the Protection of Human Subjects (45 CFR, Part 46) and supported the general ethical principles of the Declaration of Helsinki. Sixteen patients with PAH who met the World Symposium of PH Group 1 criteria and 10 healthy subjects were used in this study for plasma heme analysis. All these subjects were recruited prospectively by the University of Arizona. Written informed consent from all subjects was obtained in this study with the approval of the UA institutional human subjects review board. The venous blood was collected and stored during outpatient visits at the University of Arizona Biobank at −80°C, as previously described (9).

### Heme Preparation

Hemin (51280, Sigma-Aldrich, St. Louis, MO) solution was prepared as previously published (8). Briefly, 6.5 mg of hemin was dissolved in 5 mM NaOH to receive a 10 mM stock solution. The pH was adjusted to the physiological values (final pH 7.4), and the stock solution was diluted with 0.9% sterile saline (510223, VetOne, Boise, ID) to the final heme volume.

### Isolation and Validation of Mouse Lung Endothelial Cells

Isolation of mouse pulmonary artery endothelial cells (MPAECs) from the murine lung was performed according to the protocol described previously (10, 11). Briefly, 12–13-wk-old male WT and MKK3 KO mice (*n* = 5) were euthanized using cervical dislocation under anesthesia. The lungs were perfused with cold, sterile 0.9% saline via the right ventricle; the dissected lungs were minced and digested in 1 mg/mL collagenase type-II (LS004176, Worthington, Lakewood, NJ) and neutral protease (LS02109, Worthington, Lakewood, NJ) for 45 min at 37°C. Anti-mouse CD31 MicroBeads (130-097-418, Miltenyi Biotec, Westphalia, Germany) were used to pull down MPAEC from the digested cell suspension. Cells were plated in 12-well plates and cultured in complete endothelial cell medium (1001, ScienCell, Carlsbad, CA) containing 5% FBS and antibiotic-antimycotic (15240062, Thermo Fisher Scientific, Waltham, MA). The purity of the isolated endothelial cells was confirmed by using immunofluorescence staining with von Willebrand factor marker antibody (Supplemental Fig. S1: https://doi.org/10.6084/m9.figshare.25787664) from Proteintech, Rosemont, IL (1:100 dilution, 27186-1-AP). Cell culture was routinely monitored for the growth of contaminants. After 48–72 h in culture, the endothelial cells were used for further heme treatment and imaging experiments.

### Histopathology and Immunostaining

Pulmonary vessels of 4 μm tissue slices were dewaxed and stained with hematoxylin and eosin from the Tissue Acquisition and Cellular/Molecular Analysis Shared Resource pathology core at the University of Arizona using standardized protocols. Ten different transversely sliced pulmonary arteries (PA; diameter < 300 μm–50 μm) from animals were randomly used from the ×20 image (Leica DMI6000 Multifunction Motorized Inverted Microscope, Buffalo Grove, IL). Verhoeff–Van Gieson elastic fibers staining was done by HistoWiz Inc. (Brooklyn, NY). Immunohistochemistry was performed on 5 µm sections using standard protocols as previously described (5). Representative images were obtained from the Revolve Fluorescence Microscope (Echo, San Diego, CA) at ×40 magnification. The lung tissue sections were deparaffinized and incubated with CD68 primary antibody (1:100, 97778, Cell Signaling Technology, MA) and Dab Rabbit H1 (pH 6) for 20 min and visualized using the Leica DMI6000 Multifunction Motorized Inverted Microscope (Buffalo Grove, IL). All histology/microscopy experiments were carried out at the same time under identical conditions. The morphometric evaluation was done in a double-blinded manner using coded slides. PA occlusion score was calculated using ImageJ software (Version-1.52p National Institute of Health, Washington; http://fiji.sc/Fiji; in the public domain; 12).

Myeloperoxidase (MPO) was performed using anti-MPO antibody (1:20, ab9535, Abcam, MA). Pulmonary vessels, 5 μm tissue sections were dewaxed and stained with MPO antibody by HistoWiz Inc. (Brooklyn, NY), using standard operating procedures and a fully automated workflow system. Ten transversely sectioned images within each category per each animal (*n* = 6 per group) were randomly selected from the whole slide ×40 digitized image Aperio AT2 scanner (Leica Biosystems, IL). Isolated MPAEC from wild-type and MKK3-KO mice were used for immunocytochemistry; cells were grown in 12 well-cell culture plates with coverslips at the density of 25 × 10^3^/well as previously described (13). MPAEC were treated with (10 µM) heme for 2 h and fixed in 4% paraformaldehyde for 10 min. Cells were permeabilized with 0.2% Triton X-100 for 15 min at room temperature and 2% bovine serum albumin (37525, Thermo Fisher Scientific, Rockford, IL) for 45 min. The cells were incubated with Phospho-p38 MAP Kinase (Thr-180/Tyr-182) (1:250, PM1391, ECM Biosciences, KY) for 2 h and Anti-Mouse Ig:DyLight 488 secondary antibody (1:200, MS3011, ECM Biosciences, KY) for 30 min in dark and imaged using the Revolve Fluorescence Microscope (Echo, San Diego, CA) at ×63 magnification. The quantification was done by an investigator in a blinded grouping fashion. Eight to ten fields in each category per each animal (*n* = 6 per group) were randomly selected for quantification. The fluorescent intensity was measured using ImageJ software (12).

Ki67 staining was conducted using paraffin-embedded tissue sections that were first deparaffinized with three washes in xylene for 5 min each, followed by immersion in 100% ethanol for 4 min, and sequential rehydration in 100%, 95%, and 70% ethanol. The sections were then rinsed twice with PBS. For antigen retrieval, the slides were placed in citrate buffer at pH 6.0 and heated in a 90°C water bath for 20 min, with the temperature gradually increased. After cooling, the slides were rinsed twice in PBS. To inactivate endogenous peroxidase activity, the sections were treated with 3% hydrogen peroxide in methanol for 5 min at room temperature, followed by two PBS rinses. Blocking was performed with 0.5% BSA in PBS for 30 min at room temperature. The Ki67 antibody (ab16667, Abcam, Waltham, MA) diluted 1:100 in PBS with BSA was applied to the tissue section and incubated overnight at 4°C in a humidity chamber. The slides were then washed several times with PBS containing BSA to remove excess antibodies. A secondary anti-rabbit antibody, diluted 1:200, was applied for 45 min at room temperature. After washing with PBS-BSA, an avidin-biotin conjugate from the same secondary antibody kit was applied for 30 min at room temperature, followed by additional PBS-BSA washes. The slides were then developed using DAB (prepared by dissolving one DAB Easy Tablet in 20 mL PBS and adding 66 μL of 3% hydrogen peroxide) until the desired staining was observed, dependent on the primary antibody. The sections were washed with PBS, counterstained with Gill’s Hematoxylin for 90 s, and subsequently dehydrated through graded ethanol (70%, 95%, and 100%), cleared in xylene, and mounted with a xylene-based mounting medium (PolyMount). Imaging was performed using a Revolve Fluorescence Microscope (Echo, San Diego, CA) at ×40 magnification. Quantification was conducted by an investigator blinded to the group assignments. For each animal (*n* = 6 per group), six to eight fields per category were randomly selected for analysis. The DAB intensity was measured using ImageJ software (12). This work was supported by the Histology Core of the Indiana Center for Musculoskeletal Health at Indiana University School of Medicine and the Indiana Clinical Translational Sciences Institute.

### Western Blotting

The protein levels in pulmonary tissue and PAEC were analyzed as previously published (14–16). Briefly, the tissues were homogenized using a Fisher Brand Homogenizer-850 in a permeabilization buffer comprising Phosphatase Inhibitor Cocktail and Halt Protease (78444) from Thermo Fisher Scientific, Rockford, IL. The homogenate was centrifuged at 10,000 *g* for 10 min at 4°C, and the protein concentrations were measured by the Pierce BCA Protein Assay Kit (23225, Thermo Fisher Scientific, Burlington, ON, Canada). The samples mixed with 6X Laemmli buffer (Boston BioProducts Inc., Ashland, MA) were heated at 95°C for 5 min and run in 4–20% Mini-PROTEAN TGX Stain-Free Gels (Bio-Rad Laboratories Inc., Hercules, CA). The proteins were transferred to a PVDF membrane using a Trans-Blot Turbo transferring system (Bio-Rad Laboratories Inc., Hercules, CA). The protein membranes were blocked in 5% bovine serum albumin (37525, Thermo Fisher Scientific, Rockford, IL) for 1 h. Then, they were probed with the following antibodies: Jak2 (1:1,000, sc-390539) from Santa Cruz Biotechnology, Dallas, TX; Phospho-p44/42 MAPK (Erk1/2) (Thr202/Tyr204) (1:1,000, 9101S), p44/42 MAPK (Erk1/2) (1:1,000, 9102S), Phospho-p38 MAPK (Thr180/Tyr182) (1:1,000, 9211S), p38 MAPK (1:1,000, 9212S), zonula occludens-1 (ZO-1) (1:1,000, 5406S), p-p38 (1:1,000, 9211), MAPKAPK-2 (1:1,000, 3042), P-MAPKAPK-2 (T334) (1:1,000, 3041), HSP27 (1:1,000, 2402S), p-HSP27(1:1,000, 2401S), MKK3 (1:1,000, 8535S), Phospho-STAT3 (Ser727) (1:1,000, 9134S), STAT3 (1:1,000, 9139S), Phospho-Akt (Ser473) (1:1,000, 4060S), and Akt (1:1,000, 9272S) from Cell Signaling Technology, MA; Phospho-MKK3 (189) (1:1,000, PA5-37700) from Thermo Fisher Scientific, Rockford, IL; ICAM1 (1:1,000, 10020-1-AP), VCAM1 (1:1,000, 11444-1-AP) from ProteinTech, Rosemont, IL, and peroxisome proliferator-activated receptor gamma coactivator 1-alpha (PGC-1α) (Ab191838, 1:1,000) from Abcam, Waltham, MA. The protein bands were imaged using the chemiluminescent protocol and recorded with the ChemiDoc MP Imaging System (Bio-Rad Laboratories Inc., Hercules, CA). Analysis of the bands was done using the Image Lab software. Protein normalization was done with the total protein, determined from the stain-free gels as described previously (17). For more than one protein, some of the membranes were stripped using Restore Western Blot Stripping Buffer (21063, Thermo Fisher Scientific, Rockford, IL) and re-probed. Details of antibody validation are provided in Supplemental Table S1: https://doi.org/10.6084/m9.figshare.25814296.

### Bio-Plex Assay

Bio-Plex Pro Mouse Cytokine Group1 Panel 23-Plex (M60009RDPD, Bio-Rad Laboratories Inc., Hercules, CA) was used for the analysis of cytokines, chemokines, and growth factors in mouse plasma. Bead-based assay permitted to detect 23 different types of cytokines, chemokine, or growth factor target in a single well of a 96-well microplate. The assay was performed according to the manufacturer’s protocol. Briefly, mouse plasma was diluted two times with Bio-Plex sample diluent and added to beads covalently coupled to antibodies against 23 targets. After 30 min incubation on the shaker at room temperature, washed the beads, and biotinylated antibodies were incubated for 30 min under the same conditions. After three-times wash, streptavidin-phycoerythrin (streptavidin-PE) complex was added to bind to the biotinylated antibodies for 10 min at room temperature. Data acquisition at low photomultiplier (PMT), Reader Plate 1 (RP1) setting, and analysis data were performed using the Bio-Plex 200 System (Bio-Rad Laboratories Inc.). Below detection limit (DL) points were corrected with DL concentrations divided by two for analysis and plotting graphs.

### Apoptosis Signal-Regulating Kinase 1 Assay

Mouse ASK1 ELISA Kit (NBP2-69953, Novus Biologicals, CO) used for ASK1 assay according to the manufacturer’s instruction. WT and MKK3-KO mouse plasma samples were used for this assay, and the activity was expressed as pg/mL.

### Heme Assay

Plasma heme assay was performed in human plasma, WT, and MKK3-KO mice using the Hemin Assay Kit (ab65332, Abcam) according to the manufacturer’s instruction, and the results were expressed as in micromolar concentrations (18).

### Reactive Oxygen Species Assays

Isolated MPAECs 50,000 cells/well were plated in a 96-well plate and treated with 10 µM heme for 2 h. The Amplex Red Hydrogen Peroxide Assay Kit (A22188; Thermo Fisher Scientific, Rockford, IL) was used to detect hydrogen peroxide generation in cells grown in phenol red-free DMEM. The media was then extracted, and peroxide was determined in the media according to the manufacturer’s protocol. Results were normalized with total protein determined by the Pierce BCA Protein Assay Kit.

### Statistical Analysis

Statistical analysis was performed by using the GraphPad Prism software (version 8.3.1). The means ± standard deviation (SD) was analyzed for all samples and determined the significance by either analysis of variance (ANOVA) or *t* test. ANOVA followed by Bonferroni multiple comparison tests was used to compare between groups. The significance level was assessed by a 95% confidence interval. *P* < 0.05 was counted to be statistically significant, and Grubbs’ test was performed to identify significant outliers in the analysis.

## RESULTS

### Chronic Intravenous Supplementation of Free Heme Induces Pulmonary Hypertension

**Figure 1.**
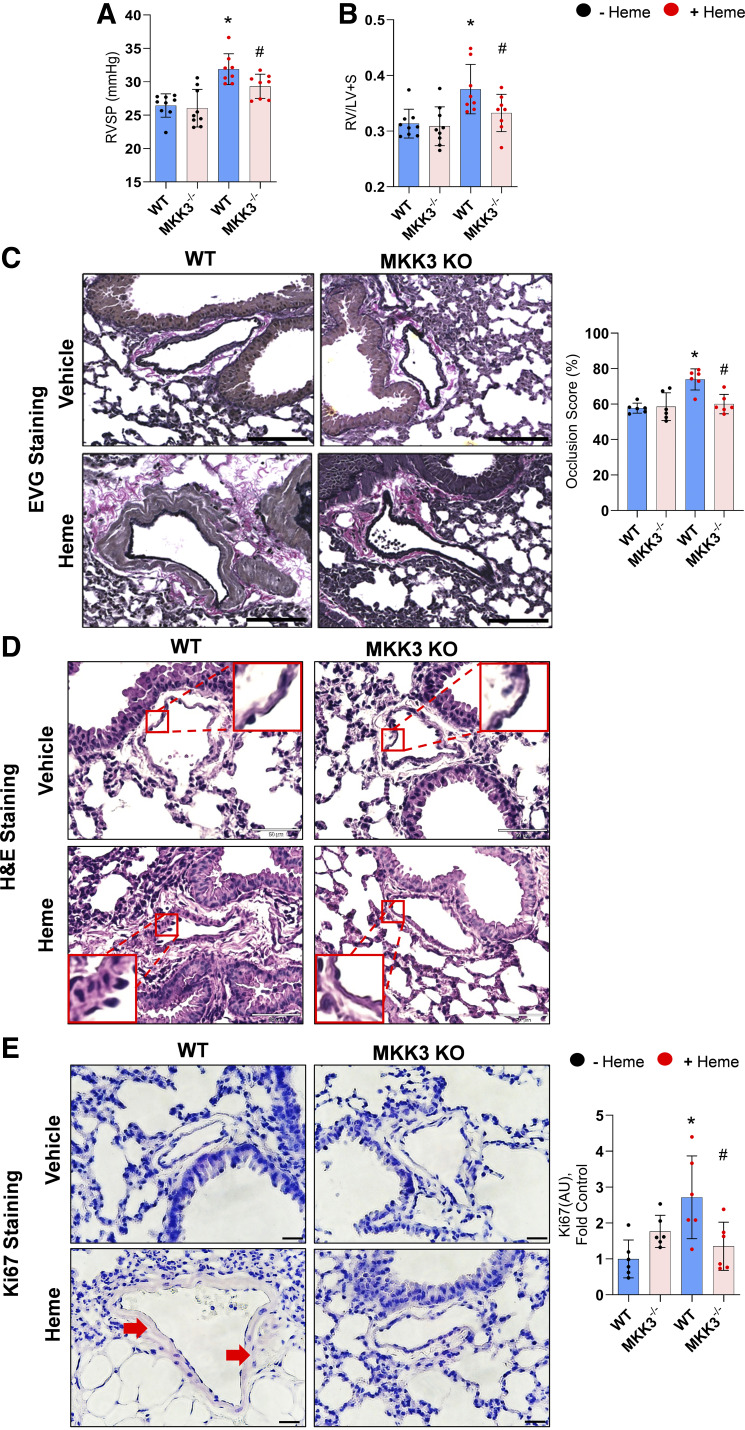
Hemodynamic alterations and histological changes. Heme-treated WT mice showed an increase in right ventricular systolic pressure (RVSP; *A*) and the ratio of right to left ventricles plus septum, the Fulton index (*B*). Histological analysis in WT + Heme group showed increased media thickening and occlusion in the pulmonary arteries. Verhoeff–Van Gieson (EVG) staining showed perivascular fibrosis and vascular remodeling in pulmonary arteries of heme-treated WT, but these alterations were significantly attenuated in the MKK3 KO model (scale is 100 µm; *C*) and hematoxylin and eosin images (scale is 50 µm; *D*). Ki67 staining showed increased proliferative remodeling in heme-treated WT lung compared with the corresponding MKK3 KO lung tissue (scale is 20 µm). *E*: red arrow indicates Ki67 staining. Images of all groups in microscopy experiments were collected at the same time under the same conditions. Black dots represent without heme treatment, and red dots represent with heme treatment. KO, knockout; WT, wild-type. Data represented as means ± SD, *n* = 6–9, *P < 0.05 vs. WT, #*P* < 0.05 vs. WT + Heme by one-way ANOVA with following Bonferroni multiple comparison test.

Although previous research from our team and others (1, 5, 13) suggests that free heme can contribute to the pathobiology of PH, its ability to initiate the disease has never been studied. To evaluate the direct impact of the circulating free heme on the pulmonary pressure and pulmonary vascular remodeling, we continually pumped heme solution at a low rate (0.85 µg/h) to the circulation of WT and MKK3 KO mice for 2 wk. The assessment of pulmonary hemodynamics confirmed a significant elevation of RVSP in WT mice treated with heme (Fig. 1*A*). Furthermore, the Fulton index [right ventricular (RV) mass adjusted to left ventricle plus septum (LV + S)], as a marker of RV hypertrophy (Fig. 1*B*). Pulmonary vascular occlusion as a sign of vascular remodeling and proliferation was also significantly increased in WT mice chronically exposed to the elevated level of circulating free heme (Fig. 1, *C*–*E*). However, dP/dT max, a measure of cardiac contractility functions, was not altered, possibly due to mild hemodynamic changes.

We have recently shown that free heme activates the MKK3/p38 MAPK signaling axis in pulmonary endothelial cells and acutely treated mice (13). To delineate the contribution of the same pathway in PH development upon prolonged exposure to heme, the chronic free heme treatment protocol was also applied to MKK3 KO mice. As expected, the MKK3 KO protected mice against heme-induced PH. Indeed, in the absence of MKK3 expression, the changes in RV pressure, RV hypertrophy, and vascular remodeling were significantly less compared with the heme-treated WTs and were statistically not different from untreated MKK3 KO except for RVSP (Fig. 1*A*).

### Activation of MKK3/p38 MAPK Pathway

**Figure 2.**
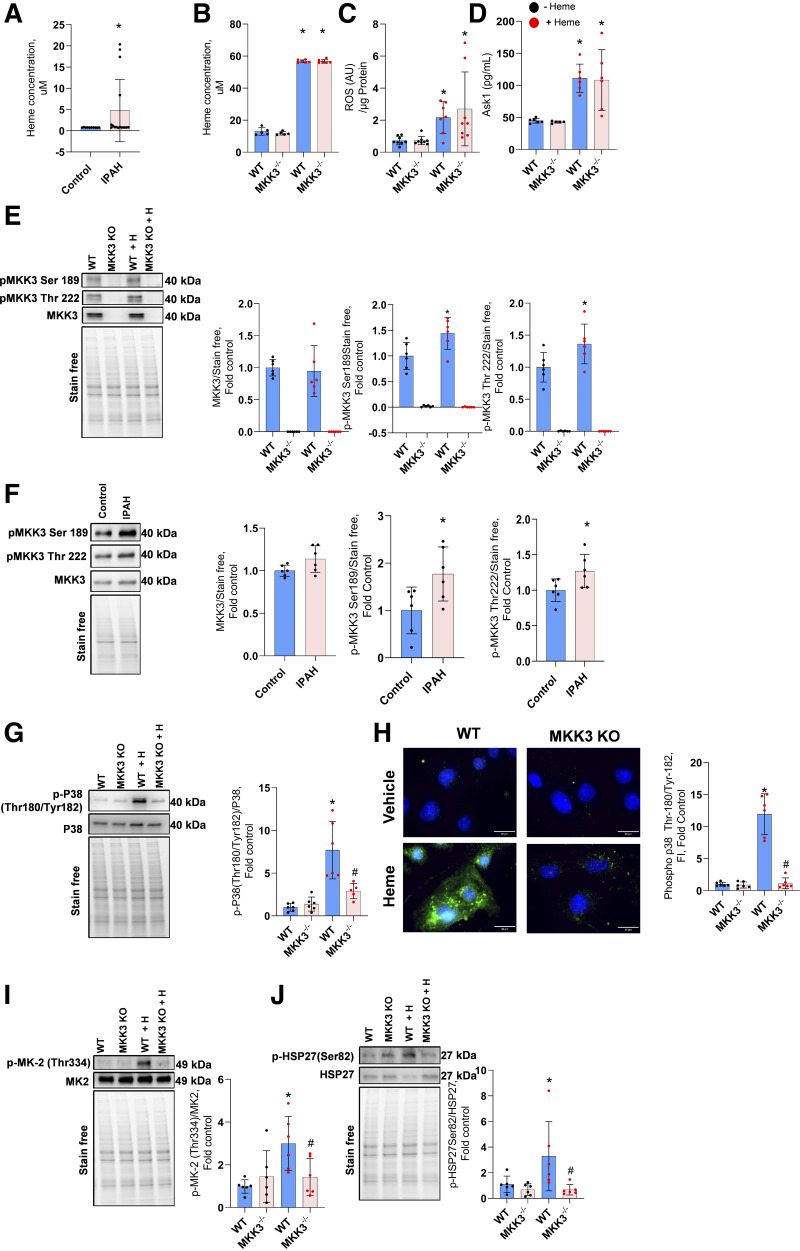
Activation of MKK3/p38 axis. The idiopathic pulmonary arterial hypertension (IPAH) population showed an increased concentration of free heme in circulation. Data represented as means ± SD fold change with the mean value of control, *n* = 16; *P < 0.05 vs. control by Mann–Whitney test (*A*). Heme treatment upregulated plasma heme levels (*B*), and it initiated mitochondrial ROS production (*C*) and Ask1 activity (*D*) similarly in WT and MKK3 KO mice. MKK3 KO mice showed no MKK3 protein expression in the lungs (*E*), but the heme treatment increased the phosphorylation of MKK3 at Ser189 and Thr222. Compared with healthy control subjects, patients with IPAH (*F*) showed increased phosphorylation of MKK3 Ser189 and Thr222, but the total MKK3 was not altered in IPAH. Data expressed as means ± SD, *n* = 6, *P < 0.05 vs. control by unpaired *t* test. Heme treatment also increased phospho-p38 (Thr180/Tyr182) in the lungs (*G*) and endothelial cells (*H*). Phospho-Thr334 MK2 (*I*) and phospho Ser82 HSP27 (*J*) were increased in the lungs, but the MKK3-KO normalized these increases. Black dots represent without heme treatment, and red dots represent with heme treatment. KO, knockout; ROS, reactive oxygen species; WT, wild-type. All other animal data represented as means ± SD, *n* = 6–10, *P < 0.05 vs. WT by one-way ANOVA with the following Bonferroni multiple comparison test.

Previously, we and others reported increased hemoglobin concentration in the plasma of patients with idiopathic pulmonary arterial hypertension (IPAH). Here, we showed an increased concentration of free heme in their circulation (Fig. 2*A*) to approximately fivefold. Importantly, heme-treated mice showed similarly increased plasma heme levels (Fig. 2*B*) after 2 wk of continuous treatment. As a result, the mitochondrial reactive oxygen species (ROS) production is activated, leading to the upregulation of ASK1, the upstream events of the MKK3 MAPK pathway in wild-type and MKK3-KO pulmonary artery endothelial cells (Fig. 2, *C* and *D*). Thus, MKK3 KO would not affect these upstream targets. As expected, MKK3 KO mice showed no MKK3 protein expression in the lungs (Fig. 2*E*). In WT animals, chronic exposure to free heme has significantly upregulated the phosphorylation level at MKK3 phosphorylation sites, Ser189 and Thr222. This result reproduces our previous study that described the upregulation of the MKK3 axis in response to the acute injection of free heme (13).

Interestingly, human lung tissue samples screening from the PHBI cohort identified that the phosphorylation of MKK3 Ser189 and MKK3 Thr222 was significantly increased in patients with idiopathic PAH (Fig. 2*F*). However, total MKK3 expression was not altered compared with the healthy control subjects. Indeed, it has been shown that MKK3-mediated inflammatory MAPK pathway activation has detrimental effects on many diseases, including cardiac hypertrophy and pulmonary fibrosis (19). Thus, the results highlight that heme-mediated effects and activation of MKK3 are significant contributors to the pathogenesis of PH in humans.

Activation of MKK3 was responsible for a robust increase (more than eightfold) in p38 MAPK phosphorylation in lung tissue from WT mice treated with heme (Fig. 2*G*). Mouse pulmonary artery endothelial cells isolated from the lungs also showed a marked increase in the phospho-p38 signal (Fig. 2*H*). Furthermore, the approximately threefold elevated phosphorylation of MK2 and HSP27 (Fig. 2, *I* and *J*), downstream of p38 activation, was found in the lungs of WT animals treated with heme. Indeed, the absence of significant changes in all three downstream targets in MKK3 KO animals provides strong evidence that MKK3 orchestrates the heme-induced effects. Considering our previous and current results, we conclude that, either acutely or chronically, free heme acts through activation of the MKK3, while the p38/MK2/HSP27 signaling pathway transduces this effect to facilitate PH pathobiology.

### Activation of Proliferative Signaling

**Figure 3.**
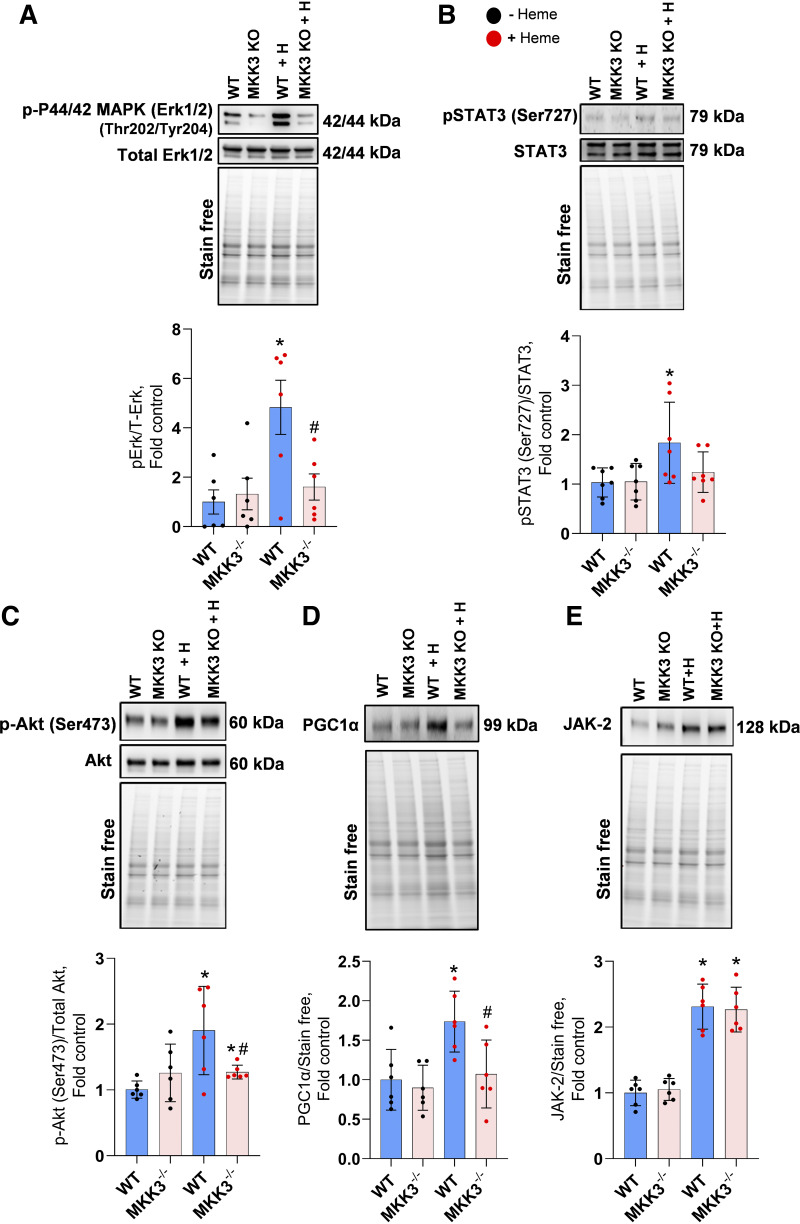
Activation of Erk/STAT/Akt/JAK signaling. Heme treatment upregulated the Erk1/2 (p44/42 MAPK; *A*), phospho STAT3 (*B*), and phospho Akt (*C*) pathways. Free heme also upregulated the PGC1α (*D*) in WT lung tissue. Notably, the absence of MKK3 attenuated the activation of Erk, Stat, Akt, and PGC1α. JAK2, upstream of p38, is activated by heme but was not affected in the MKK3 KO (*E*). Black dots represent without heme treatment, and red dots represent with heme treatment. KO, knockout; WT, wild-type. Data represented as means ± SD, *n* = 6, *P < 0.05 vs. WT, #*P* < 0.05 vs. WT + Heme by one-way ANOVA with following Bonferroni multiple comparison test.

There are several hallmarks of increased p38 MAPK activity, including activation of proliferative signaling pathways. To evaluate whether chronic exposure to heme activated this signaling in the lungs, we measured the activities of Erk1/2, STAT3, Akt, and JAK2, which are part of the MAPK signaling network (20, 21). We found that Erk1/2, STAT3, and Akt activities were significantly upregulated by heme stimulation but remained unchanged in the absence of MKK3 expression (Fig. 3, *A*–*C*). Furthermore, heme-activated signaling cascades increased PGC-1α expression (Fig. 3*D*), a key transcriptional coactivator and the regulator of mitochondrial biogenesis under stress conditions (15), in the MKK3-dependent manner. On the contrary, JAK2 increased in response to heme treatment in both animal groups, WT and MKK3 KO (Fig. 3*E*), due to its upstream MKK3/p38 signaling position. We conclude that heme acts primarily through the MKK3-dependent pathways, although it also activates some MKK3-independent signaling. Activating proliferative cell pathways by free heme could be responsible for heme-induced pulmonary vascular remodeling (Fig. 1*E*).

### Heme-Induced Endothelial Barrier Disruption and Inflammatory Cell Infiltration

**Figure 4.**
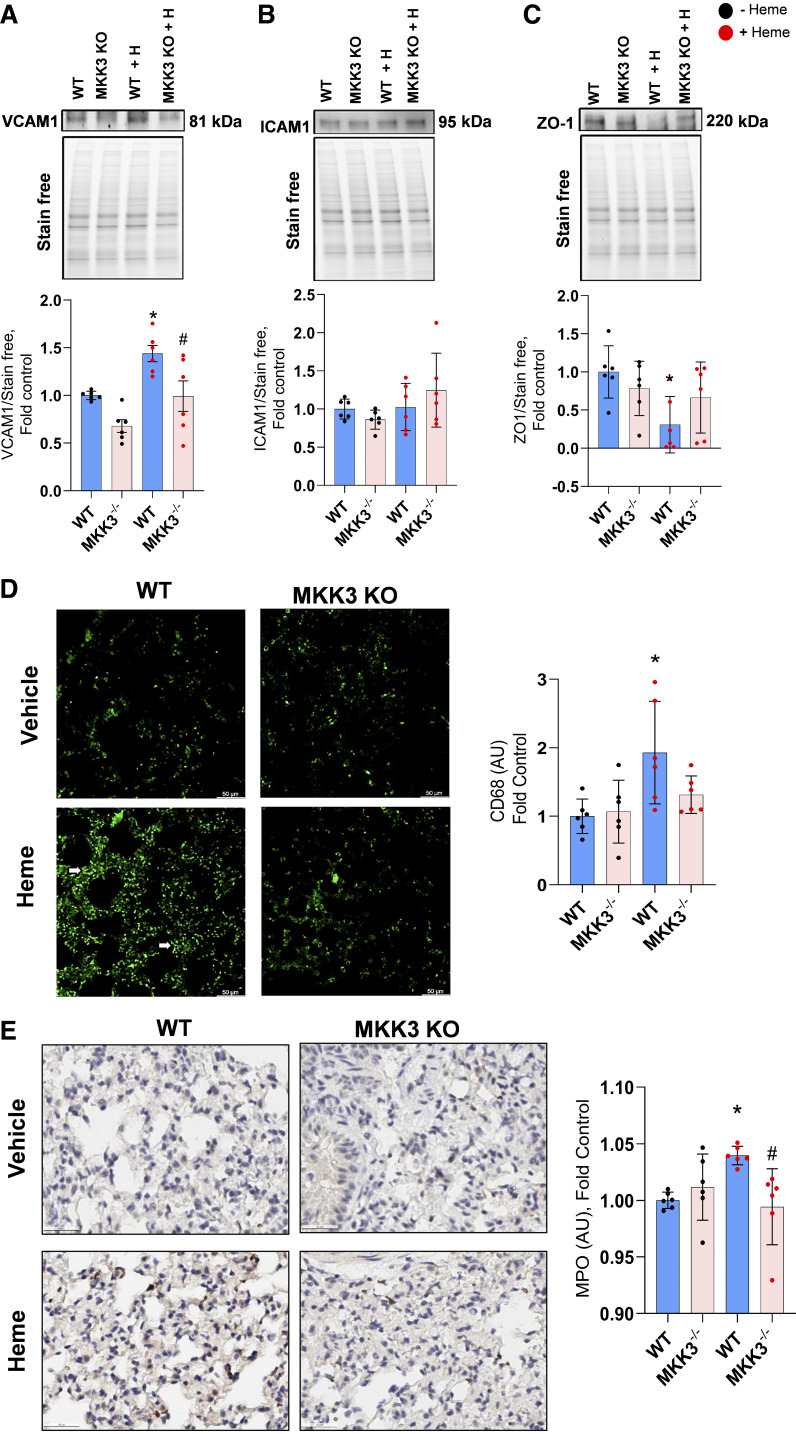
Barrier dysfunction and lung inflammatory cell infiltration. Heme treatment increased the expression of VCAM1 (*A*) in WT but not in MKK3 KO mice. ICAM1 expression was not altered with 2 wk of heme treatment (*B*). MKK3 KO mice have protected the downregulation of tight junction protein zonula occludens-1 (ZO-1; *C*) with heme treatment. Heme-induced increase in CD-68 and myeloperoxidase-positive cells (*D* and *E*) was significantly attenuated in the MKK3 KO mice (scale is 50 µm). Black dots represent without heme treatment, and red dots represent with heme treatment. KO, knockout; WT, wild-type. Data represented as means ± SD, *n* = 6, *P < 0.05 vs. WT, #*P* < 0.05 vs. WT + Heme by one-way ANOVA with following Bonferroni multiple comparison test.

Another well-established downstream effect of p38 MAPK activation is increased inflammatory response with enhanced expression of endothelial adhesion molecules and elevated endothelium permeability for inflammatory cells. Indeed, we found that chronic heme treatment stimulated the expression of the VCAM1 adhesion molecule (Fig. 4*A*), which increases the binding of circulating inflammatory cells to the endothelium (22). Importantly, MKK3 KO mice showed no significant increase in VCAM1 expression in response to free heme. In contrast, ICAM1, epithelial by its origin, the intracellular adhesion molecule, did not respond to the free heme treatment in either group (Fig. 4*B*).

The increased endothelial/inflammatory cell interaction is an essential first step of inflammatory cell infiltration. However, it is insufficient and requires a disturbed endothelial barrier. Indeed, we found that the expression of ZO-1, a critical tight junction protein, was significantly decreased in WT mice (Fig. 4*C*). These changes in ZO-1 expression correlated well with our previously published reports about the ability of free heme to induce endothelial barrier disruption in the acute regimen. Moreover, the absence of changes in the ZO-1 expression levels in MKK3 KO mice strongly supports the involvement of MKK3 in endothelial barrier dysfunction.

As expected, based on increased VCAM1 and reduced ZO-1 expression, the levels of CD68 and MPO staining as an indicator of inflammatory cell infiltration were markedly increased in heme-treated WT animals. In contrast, heme-treated MKK3 KOs were not significantly different from the untreated mice (Fig. 4, *D* and *E*). This important discovery confirms the heme-induced significant inflammatory and immune response in the lungs mediated through the MKK3/p38 pathway.

### Free Heme-Induced Cytokine Storm

**Figure 5.**
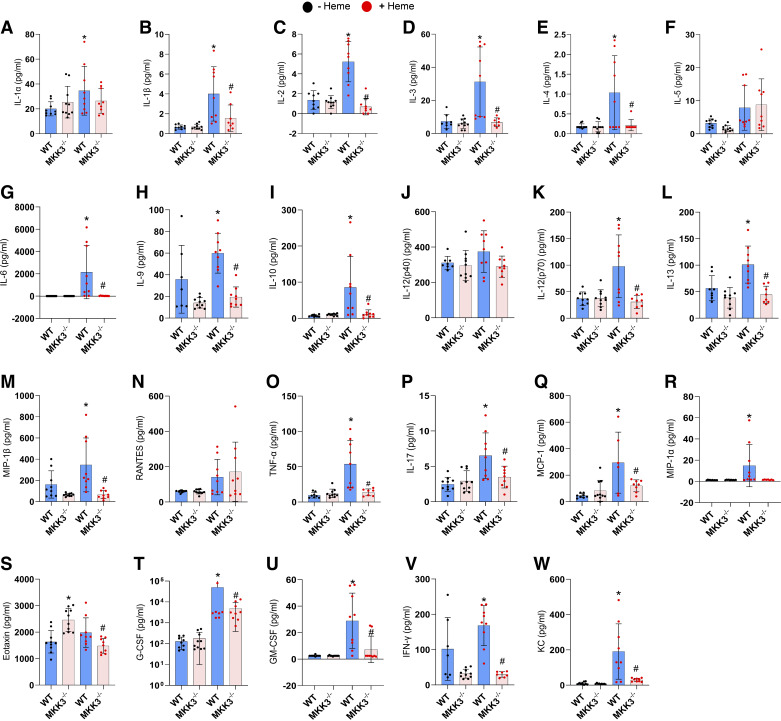
Heme-induced cytokine storm. Acute heme treatment for 24 h resulted in an inflammatory cytokine storm with a surge of 19 cytokines from the following 23 cytokines. IL-1α (*A*), IL-1β (*B*), IL-2 (*C*), IL-3 (*D*), IL-4 (*E*), IL-5 (*F*), IL-6 (*G*), IL-9 (*H*), IL-10 (*I*), IL-12 (p40; *J*), IL-12 (p70; *K*), IL-13 (*L*), IL-17 (*M*), MCP-1 (*N*), MIP-1α (*O*), MIP-1β (*P*), RANTES (*Q*), TNF-α (*R*), Eotaxin (*S*), G-CSF (*T*), GM-CSF (*U*), IFNγ (*V*), and KC (*W*). MKK3 KO model significantly attenuated the heme-induced cytokine storm. Black dots represent without heme treatment, and red dots represent with heme treatment. KO, knockout; WT, wild-type. Data represented as means ± SD, *n* = 6–8, *P < 0.05 vs. WT, #*P* < 0.05 vs. WT + Heme by one-way ANOVA with the following Bonferroni multiple comparison test.

The pleiotropic cascade induced by the p38 also includes the regulation of inflammatory cytokine production (23–25). Therefore, in this study, we investigated whether the heme-activated MKK3/p38 pathway increases the levels of circulating cytokines in WT and MKK3 KO mice. As the cytokine storm in patients with inflammatory lung diseases is described as a part of the acute hyperimmune response (26), in this experiment, we collected the blood samples 24 h after the bolus heme injection. We found that in WT, the early inflammatory response to the free heme injection involves a robust increase in the expression of 19 cytokines out of 23 analyzed (Fig. 5, *A*–*W*). However, the prolonged heme treatment could not maintain the increased cytokine profile over time (Supplemental Fig. S2, https://doi.org/10.6084/m9.figshare.25787664), possibly due to the resolution of the initial inflammation. For most activated cytokines, the absence of the MKK3 protein was protective, as the profile of MKK3 KO mice showed no or mild increase in the cytokine levels upon the heme treatment. We conclude that free heme, acting primarily through an MKK3/p38 axis, induces a severe cytokine storm. Cytokine-mediated inflammation is considered one of the significant contributors to the development of PH (27). Therefore, the severe upregulation of cytokine production in response to free heme could play an essential role in the discovered causative role of heme in PH initiation.

## DISCUSSION

The significantly higher prevalence of PH in patients with hemolytic disorders compared with the general population (28) highlights the potential contribution of hemolytic products to the pathogenesis of this PH. Moreover, hemolysis increases the severity and impairs the outcome of other lung diseases (29). With the COVID-19 pandemic, this becomes especially evident in patients with COVID-19-induced acute respiratory distress syndrome. Higher hemolytic markers are associated with a significantly more extended hospital stay and a poor overall prognosis (30). Currently, this effect of hemolysis is mainly viewed through the increased predisposition of patients with low hemoglobin to hypoxia. However, immunogenic properties of free heme point toward its direct contribution to the pathology of hemolysis-associated conditions through activation of the innate immune system (31, 32). Indeed, COVID-19-associated hemolytic anemia was described as occurring within a timeframe compatible with the cytokine storm (31). Thus, the signaling induced by the free heme could be a part of life-threatening immune system overdrive in patients with COVID-19. Furthermore, in this study, we confirmed that the increased levels of circulating free heme directly contribute to the cytokine storm associated with PH.

Aside from the severe hemolytic disorders secondary to inherited conditions (hemoglobinopathies, glucose-6-phosphate dehydrogenase) or pyruvate kinase deficiencies), immune-mediated anemia, or anemias due to the physical damage of RBC (by artificial heart valve or during hemodialysis), many other factors are reported to be associated with a high risk of intravascular hemolysis (33). However, this type of hemolysis could remain relatively mild and, in many cases, unrecognized. This includes infections, exposure to drugs and toxins, thermal or osmotic injury, strenuous exercises or trauma, vitamin E or B12 deficiency, pregnancy, liver diseases, etc. Notably, many of these conditions are also known as the risk factors for PAH. The increased prevalence of PAH upon exposure to these factors could be linked to the risk of hemolysis. Indeed, we and others have previously reported that patients with PAH without a known hemolytic history still have increased levels of circulating free hemoglobin compared with healthy subjects (5, 34). During the sickle cell crisis, plasma levels of free Hb level were reported to be as high as 25 μM (35). Moreover, there was a significant correlation between the level of circulating free hemoglobin and PAH severity (36, 37). Repetitive hemolysis can induce angioproliferative vasculopathy and increased right ventricular pressure (37). Chronic hemolysis in sickle cell disease reduced the heme transport protein hemopexin and increased heme in circulation induced oxidative stress (38, 39).

However, although reported in many studies, the association between hemolytic events and PH initiation was never confirmed directly. Therefore, we designed a model of chronic continuous intravascular free heme supplementation. Using this model, we tested whether the increase in circulating heme for 2 wk was sufficient to induce PH in WT mice. The result fully confirms the pathogenic role of heme, as the continuous heme supplementation was sufficient to significantly elevate RVSP and induce RV hypertrophy and pulmonary artery remodeling. Moreover, by including the MKK3 KO mice in this study, we discovered that the activation of the MKK3/p38 axis is the central event in the heme-mediated signaling cascade. Previously, Görlach et al. (40) reported that the MKK3/p38 axis activation in thrombin treatment is a cause of pulmonary artery smooth muscle cell proliferation. Moraes et al. (41) identified that heme treatment can enhance the proliferation of smooth muscle cells. The pleiotropic effect, initiated by MKK3/p38 axis, included cytoskeletal reorganization, cell proliferation, endothelial barrier dysfunction, and severe inflammatory response.

Such a multicomponent response could explain why the increased levels of circulating free heme alone induce the PH phenotype in a relatively short time frame. Indeed, the initial cytokine production on heme treatment triggered alterations in the pulmonary vasculature. Besides, the surge in the circulating cytokines, found as a part of the early response to the free heme, could initiate a continuous feedforward pathogenic loop. Indeed, cytokines such as TNFα and IL-1β stimulate the activity of the p38 pathway (42, 43). Activation of p38 was also described as an early response to IL-17 and hemopoietic growth factors, including GM-CSF and IL-3 (44, 45). Stimulated p38 signaling, in turn, promotes a further increase in TNFα, IL-1β, and IL-6 (46). IL-12 or the synergetic action of IL-12/IL-2 and IL-4/IL-2 stimulate T cells in a p-38-dependent manner (47–49). This p38-induced signaling cascade in T helper type 2 (Th2) cells is critical for the expression of IL-4, IL-5, IL-10, and IL-13, stimulation of B cells, eosinophil-mediated, and mast-cell-mediated immune responses (50). Thus, p38 signaling is recognized as a critical regulator of the effector function of Th2 cells and plays a central role in orchestrating the immune system (51). Th2-mediated response, in turn, controls humoral immunity, is involved in autoimmune and chronic inflammation (52, 53), and is known as an essential mediator of pulmonary arterial remodeling in PH (54, 55). Previous studies reported that hemolysis releases increased hemoglobin can trigger inflammation, and macrophage release in circulation can induce vascular remodeling (36, 56, 57). Notably, we discovered that the cytokines discussed earlier as the activators or effectors of p38 signaling were significantly elevated in response to the free heme. The previous studies and our current results make us propose the novel role of the p38 pathway in the immune response initiated by the free heme. Indeed, we confirmed that once the MKK3/p38 signaling axis was abolished, the heme-mediated cytokine storm was utterly prevented.

**Figure 6.**
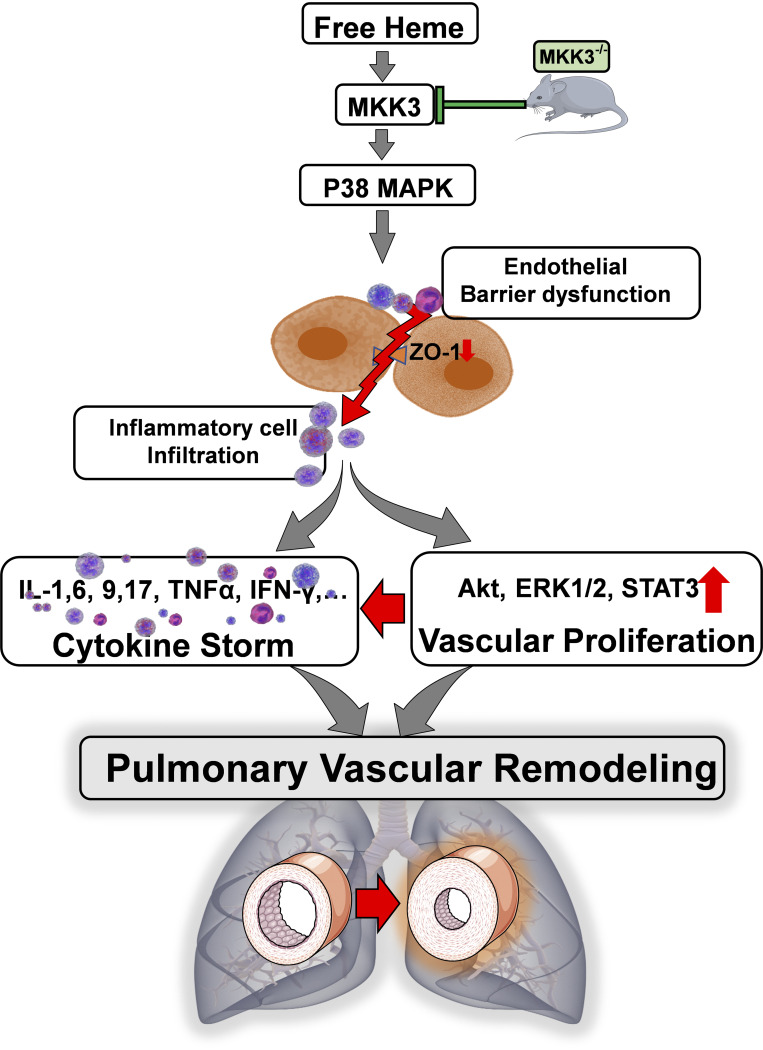
Heme-induced inflammatory and proliferative lung diseases are mediated through MKK3/P38 axis. Free heme-activated MKK3 triggered the p38 pathway, leading to the initiation of inflammatory and proliferative signaling cascades ERK1/2/STAT3/Akt. Moreover, heme treatment-induced barrier dysfunction decreases the expression of tight junction proteins (ZO-1) and facilitates inflammatory cell infiltration. However, the absence of MKK3 attenuated heme-induced inflammatory cell infiltration, cytokine storm, and pulmonary vascular remodeling.

In conclusion, this study provides clear evidence of the pathogenic role of the free heme in PH development. Furthermore, it suggests the critical importance of heme-mediated signaling in inflammatory pulmonary diseases associated with severe or mild hemolytic conditions. The underlined molecular pathways involve the MKK3/p38 signaling cascade that mediates pulmonary endothelial dysfunction, vascular remodeling, and sustained inflammatory response (Fig. 6). This discovery highlights the MKK3 as a novel, attractive therapeutic target for PH and other lung inflammatory diseases linked to hemolytic anemia.

Limitations of the present study: Although ROS is shown to be a critical factor in heme-induced disease, this study does not explicitly demonstrate whether it is mitochondrial or cytosolic ROS. Furthermore, our study did not include an assessment of endotoxin levels in the mice postexperiment to evaluate their potential impact on cytokine production. Despite this, we did not anticipate significant endotoxin contamination due to the porcine origin of the heme used. Supporting this, the study by Thomas et al. (58) reported that heme from the same source was confirmed to be endotoxin-free. Additionally, it is important to note that our current study did not include an investigation into the effects of hemopexin treatment on the interaction between heme and cellular or tissue responses. Future research could benefit from exploring how hemopexin influences heme metabolism and its associated biological effects, as this could provide valuable insights into the broader context of heme biology and its regulatory mechanisms.

## DATA AVAILABILITY

Data will be made available upon reasonable request.

## SUPPLEMENTAL MATERIAL

10.6084/m9.figshare.25787664.v1 Supplemental Figs. S1 and S2: https://doi.org/10.6084/m9.figshare.25787664.v1.

10.6084/m9.figshare.25814296.v1 Supplemental Table S1: https://doi.org/10.6084/m9.figshare.25814296.v1.

## GRANTS

This work was supported by NIH Grants R01HL133085 (to O.R.), R01HL160666 (to O.R.), R01HL151447 (to R.R.), R01HL132918 (to R.R.), and NIH K99HL171869 (to J.J.). The AHA postdoctoral fellowship 831538 (to M.V.V.), 834220 (to J.J.), and AHA Transformational award 969574 (to O.R.). Data/tissue samples provided by PHBI under the Pulmonary Hypertension Breakthrough Initiative (PHBI). Funding for the PHBI is provided under an NHLBI R24 Grant, #R24HL123767, and by the Cardiovascular Medical Research and Education Fund. The graphical abstract was created with BioRender.com.

## DISCLOSURES

No conflicts of interest, financial or otherwise, are declared by the authors.

## AUTHOR CONTRIBUTIONS

M.V.V., F.R., O.R., and R.R. conceived and designed research; M.V.V., J.J., and D.B. performed experiments; M.V.V., J.J., and D.B. analyzed data; M.V.V., J.J., O.R., and R.R. interpreted results of experiments; M.V.V. and J.J. prepared figures; M.V.V., J.J., O.R., and R.R. drafted manuscript; M.V.V., F.R., O.R., and R.R. edited and revised manuscript; M.V.V., F.R., O.R., and R.R. approved final version of manuscript.
